# Prognostic value of the log odds of negative lymph nodes/T stage ratio (LONT) in postoperative esophageal cancer: a SEER-based study

**DOI:** 10.3389/fonc.2025.1619106

**Published:** 2025-08-20

**Authors:** Yanhong Lin, Dinghang Chen, Jieming Lu, Yicheng Huang, Ziyang Han, Mingqiang Kang

**Affiliations:** ^1^ Department of Thoracic Surgery, Fujian Medical University Union Hospital, Fuzhou, Fujian, China; ^2^ Key Laboratory of Cardio-Thoracic Surgery, Fujian Medical University, Fuzhou, Fujian, China; ^3^ Key Laboratory of Gastrointestinal Cancer, Fujian Medical University, Ministry of Education, Fuzhou, Fujian, China; ^4^ Fujian Key Laboratory of Tumor Microbiology, Department of Medical Microbiology, Fujian Medical University, Fuzhou, Fujian, China; ^5^ Clinical Research Center for Thoracic Tumors of Fujian Province, Fuzhou, Fujian, China

**Keywords:** esophageal cancer, LONT, RSF, postoperative, SEER

## Abstract

**Introduction:**

Surgery remains the primary treatment for patients with esophageal cancer (EC), yet postoperative prognosis is often unsatisfactory. Accurate prediction of cancer-specific survival (CSS) can assist clinicians in personalized treatment planning. This study aimed to develop an interactive web-based tool to estimate CSS in patients with T1~3N0~2M0 EC after surgery, based on the log odds of negative lymph nodes/T stage ratio (LONT).

**Methods:**

A total of 2,221 patients with T1~3N0~2M0 EC were identified from the Surveillance, Epidemiology, and End Results (SEER) database. Patients were randomly divided into training and testing sets. Univariate Cox regression analysis was conducted to identify factors associated with CSS. Cox regression and random survival forest (RSF) models were used to compare the predictive performance of LONT and N stage. Model performance was evaluated using receiver operating characteristic (ROC) curves, decision curve analysis (DCA), and calibration curves. An interactive web-based tool was then constructed for individualized survival prediction.

**Results:**

Univariate analysis revealed that age, sex, T stage, N stage, chemotherapy, and LONT were significantly associated with CSS. ROC curve comparisons showed that LONT outperformed N stage in predictive accuracy, particularly for 1-year CSS. DCA and calibration curves indicated that the model had high predictive accuracy in both training and testing sets.

**Discussion:**

The developed interactive web-based tool provides effective estimation of 1-, 3-, and 5-year CSS, as well as survival trends, in postoperative patients with T1~3N0~2M0 EC. This tool may aid clinical decision-making by enabling more accurate individualized prognosis prediction.

## Introduction

Epidemiological studies indicate that esophageal cancer (EC) ranks seventh in global cancer incidence and sixth in cancer-related mortality (1). Early-stage EC often presents with nonspecific symptoms, resulting in many patients being diagnosed at advanced stages and missing the optimal window for surgical treatment (2). Surgery remains the primary treatment for EC, however, the survival rate for patients undergoing surgery is still suboptimal (3). Therefore, identifying reliable prognostic factors for surgically treated EC is essential. The American Joint Committee on Cancer (AJCC) TNM stage system, particularly the pT and pN classifications, is widely used to stage tumors. However, the N stage is based solely on the number of positive lymph nodes (PLN), without accounting for the number of negative lymph nodes (NLN), which may limit its ability to accurately reflect tumor burden and affect staging precision and comparability (4–6). Studies have shown that the number of NLN holds prognostic significance in EC and serves as an independent predictor in patients undergoing curative esophagectomy (7). Additionally, T stage is strongly correlated with both prognosis and tumor biology (8, 9). However, few prognostic indicators simultaneously incorporate both T stage and NLN count. In recent years, the log odds of negative lymph nodes/T stage ratio (LONT), a metric combining T stage and NLN data, has emerged as a superior predictor of survival compared to T, N, or TNM stage alone (10). LONT has demonstrated strong prognostic utility in several cancers, including colorectal (10, 11), thyroid (12), and bladder cancers (13). However, its prognostic value in EC remains largely unexplored. Therefore, this study investigates the prognostic significance of LONT in postoperative patients with T1~3N0~2M0 EC and introduces a web-based dynamic survival prediction tool based on LONT to enhance personalized prognosis assessment.

## Methods

### Data sources and patients

**Figure 1:**
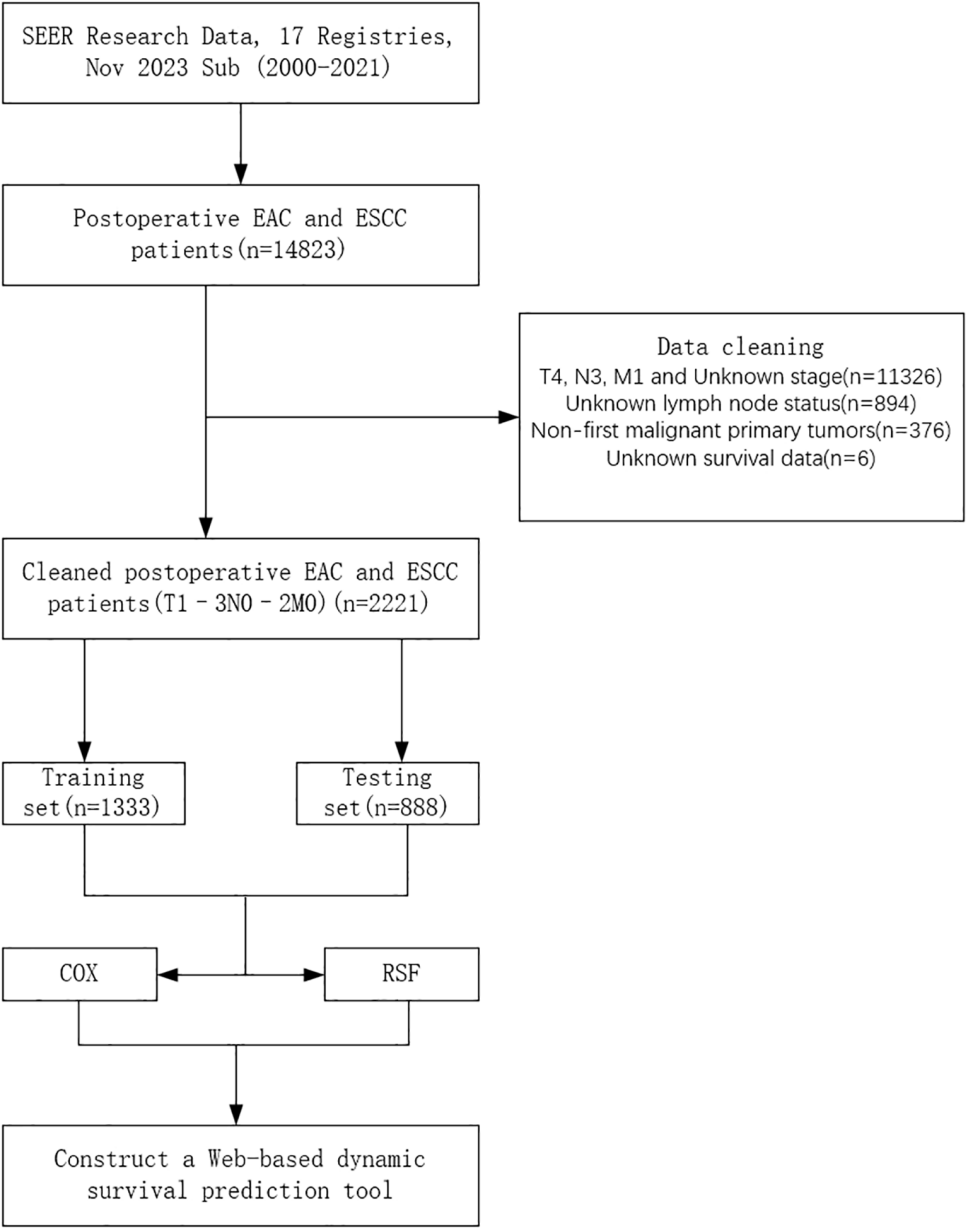
Flowchart of patient selection. EAC, Esophageal Adenocarcinoma; ESCC, Esophageal Squamous Cell Carcinoma; COX, Cox Proportional Hazards Model; RSF, Random Survival Forest.

The patient data used in this study were obtained from the Surveillance, Epidemiology, and End Results (SEER) database, a population-based cancer registry covering approximately 34.6% of the U.S. population. Using SEER*Stat software (version 8.4.4), we retrospectively extracted data from 2000 to 2021 across 17 registries. Eligible cases included patients with primary site codes C15.3–C15.5 (upper to lower esophagus), histological types 8070/3 (squamous cell carcinoma) or 8140/3 (adenocarcinoma), clinical stages T1~3N0~2M0, and who underwent surgical treatment. The inclusion criteria were: (1) esophageal cancer (EC) patients who received surgical treatment; (2) pathologically confirmed T1~3N0~2M0 adenocarcinoma or squamous cell carcinoma; and (3) EC as the only primary tumor at diagnosis. The exclusion criteria were: (1) unknown or incomplete pathological data; (2) missing lymph node information; (3) EC not being the first primary tumor; and (4) missing survival data. A total of 2,221 patients from the SEER database met the criteria and were included in this retrospective study. Ethical approval and informed consent were not required for this study, as the SEER database provides de-identified, publicly available data and is therefore exempt from institutional review board (IRB) oversight. The complete data selection process is illustrated in **Figure 1**.

### Selection and definition of variables

The variables extracted from the SEER database for this study included age, sex, histology, tumor location, T stage, N stage, chemotherapy, and log odds of negative lymph nodes/T stage ratio (LONT). LONT was defined as log((NLN +1)/T stage). NLN was calculated by subtracting the number of positive lymph nodes (PLN) from the number of examined lymph nodes (ELN), and one was added to avoid division by zero. T1, T2, and T3 stages were assigned numeric values of 1, 2, and 3, respectively. The optimal cutoff values for age and LONT were determined using X-tile software and were identified as 52 and 71 years for age (**Supplementary Figure S1**), and 1.0 and 2.3 for LONT (**Supplementary Figure S2**).

### Cox proportional hazards and random survival forest models

The Cox proportional hazards model is one of the most widely used tools in survival analysis. In this study, we performed univariate Cox regression analyses for all variables and considered those with a p-value < 0.05 as prognostic factors for cancer-specific survival (CSS). We then conducted two separate multivariate Cox regression analyses: Model 1 focused on LONT, while Model 2 emphasized N stage. Random survival forest (RSF) was constructed using an ensemble of binary decision trees and are effective in identifying predictors closely associated with time-to-event outcomes. RSF introduces dual randomization during model construction: bootstrapping is used to sample the data, and a random subset of covariates is selected at each node for splitting (14). In this study, RSF models were trained based on Model 1 (LONT) and Model 2 (N stage), each including seven variables. The terminal node size was set to 10, and two variables were randomly selected for splitting at each node. The RSF models offered an effective method for systematically identifying key clinical factors associated with CSS, providing a theoretical foundation for personalized survival prediction and risk stratification.

### Statistical analysis

In this study, we performed comprehensive statistical analyses using R software (version 4.4.1) and X-tile software (version 3.6.1). X-tile was used to determine the optimal cutoff values for age and LONT. Demographic and clinical variables were analyzed using the chi-square test, and baseline characteristics were reported as counts and percentages (n, %). The primary endpoint was CSS. Univariate and multivariate Cox regression analyses were performed using the “survival” and “autoReg” packages in R, with multivariate models developed separately based on LONT and N stage. ROC curves for Cox and RSF models were generated using the “pROC” and “timeROC” packages to compare the predictive performance of Model 1 (LONT) and Model 2 (N stage). The RSF model was constructed using the “randomForestSRC” package. Decision curve analysis (DCA) and calibration curves were plotted using the “ggDCA” and “rms” packages, respectively. Calibration curves assessed the agreement between predicted probabilities and actual outcomes, while DCA evaluated the net clinical benefit across a range of risk thresholds, highlighting the model’s clinical utility. Survival curves were plotted using the “survminer” and “survival” packages. Finally, an interactive web-based tool was developed based on LONT to enable personalized survival prediction.

## Results

### Baseline characteristics

Based on the inclusion and exclusion criteria, a total of 2,221 postoperative esophageal cancer (EC) patients with T1~3N0~2M0 stage were included from the Surveillance, Epidemiology, and End Results (SEER) database. The patients were randomly assigned to training and testing sets in a 6:4 ratio. The demographic and clinicopathological characteristics of the training and testing sets are summarized in **Table 1**. In the overall set, 84.4% of the patients were male, and 72.2% were aged between 52 and 71 years. Additionally, the majority of patients were diagnosed with esophageal adenocarcinoma (EAC) (82.9%), had tumors located in the lower third of the esophagus (87.0%), were classified as T3 stage (56.7%), or had N0 stage (46.1%). Regarding treatment, a higher proportion of patients received chemotherapy (77.2%).

**Table 1:** Demographic and clinicopathological characteristics of the training and testing sets.

Variable	Overall	Training set	Testing set	P-value
	(n=2221)	(n=1333)	(n=888)	
Age (n, %)				
<52	236 (10.6)	135 (10.1)	101 (11.4)	0.63
52-71	1604 (72.2)	970 (72.8)	634 (71.4)	
>71	381 (17.2)	228 (17.1)	153 (17.2)	
Sex (n, %)				
Male	1874 (84.4)	1124 (84.3)	750 (84.5)	0.977
Female	347 (15.6)	209 (15.7)	138 (15.5)	
Histology (n, %)				
AC	1841 (82.9)	1113 (83.5)	728 (82.0)	0.384
SCC	380 (17.1)	220 (16.5)	160 (18.0)	
Tumor location (n, %)				
Upper third of esophagus	32 (1.4)	14 (1.1)	18 (2.0)	0.155
Middle third of esophagus	257 (11.6)	152 (11.4)	105 (11.8)	
Lower third of esophagus	1932 (87.0)	1167 (87.5)	765 (86.1)	
T stage (n, %)				
T1	575 (25.9)	343 (25.7)	232 (26.1)	0.886
T2	387 (17.4)	229 (17.2)	158 (17.8)	
T3	1259 (56.7)	761 (57.1)	498 (56.1)	
N stage (n, %)				
N0	1024 (46.1)	611 (45.8)	413 (46.5)	0.824
N1	897 (40.4)	545 (40.9)	352 (39.6)	
N2	300 (13.5)	177 (13.3)	123 (13.9)	
Chemotherapy (n, %)				
Yes	1714 (77.2)	1021 (76.6)	693 (78.0)	0.457
No	507 (22.8)	312 (23.4)	195 (22.0)	
LONT (n, %)				
<1.0	350 (15.8)	206 (15.5)	144 (16.2)	0.27
1.0-2.3	1259 (56.7)	743 (55.7)	516 (58.1)	
>2.3	612 (27.6)	384 (28.8)	228 (25.7)	

LONT, Log Odds of Negative Lymph Nodes/T stage ratio.

### identification of independent Prognostic Factors for CSS

**Figure 2:**
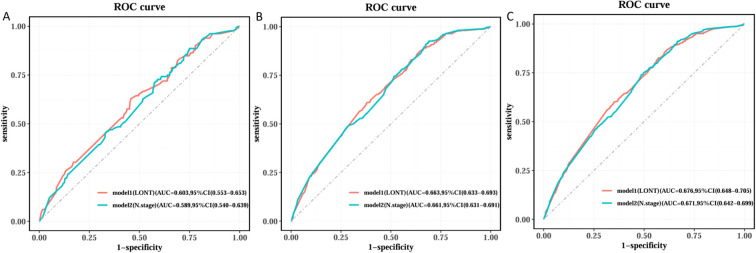
Comparison of ROC curves among different Cox models. **(A)** Time of the ROC in 1 year, **(B)** Time of the ROC in 3 years and **(C)** Time of the ROC in 5 years.

We used Cox regression analysis to identify independent prognostic factors for CSS in EC patients. Univariate Cox analysis revealed that age, sex, T stage, N stage, chemotherapy, and LONT were significantly associated with CSS (**Table 2**). Subsequently, we performed two multivariate Cox regression analyses based on LONT and N stage, respectively (**Table 3**). ROC curve analysis of the multivariate Cox models showed that Model 1 (LONT) demonstrated superior predictive performance compared to Model 2 (N stage), with AUCs of 0.603 at 1 year, 0.663 at 3 years, and 0.676 at 5 years (**Figure 2**).

**Table 2:** Univariate Cox proportional hazards regression analysis of esophageal cancer prognosis.

Variable	HR (univariable)
Age	
<52	Ref
52-71	1.19 (0.90-1.59, p=0.225)
>71	1.41 (1.01-1.97, p=0.041)
Sex	
Male	Ref
Female	0.78 (0.61-0.99, p=0.041)
Histology	
AC	Ref
SCC	1.00 (0.80-1.24, p=0.980)
Tumor location	
Upper third of esophagus	Ref
Middle third of esophagus	1.32 (0.54-3.28, p=0.544)
Lower third of esophagus	1.19 (0.49-2.88, p=0.695)
T stage	
T1	Ref
T2	1.83 (1.36-2.45, p<.001)
T3	2.84 (2.25-3.58, p<.001)
N stage	
N0	Ref
N1	1.88 (1.57-2.26, p<.001)
N2	2.40 (1.89-3.05, p<.001)
Chemotherapy	
Yes	Ref
No	0.40 (0.32-0.51, p<.001)
LONT	
<1.0	Ref
1.0-2.3	0.66 (0.54-0.81, p<.001)
>2.3	0.33 (0.25-0.42, p<.001)

LONT:Log Odds of Negative Lymph Nodes/T stage ratio.

**Table 3:** Multivariate Cox regression analysis for CSS.

Variable	Model 1 (LONT)	Model 2 (N stage)
Age		
<52	Ref	Ref
52-71	1.18 (0.89-1.57, p=0.261)	1.17 (0.88-1.56, p=0.281)
>71	1.56 (1.12-2.18, p=0.009)	1.51 (1.08-2.11, p=0.015)
Sex		
Male	Ref	Ref
Female	0.77 (0.60-0.99, p=0.042)	0.75 (0.59-0.97, p=0.028)
Histology		
AC	Ref	Ref
SCC	0.93 (0.71-1.20, p=0.563)	0.95 (0.73-1.23, p=0.692)
Tumor location		
Upper third of esophagus	Ref	Ref
Middle third of esophagus	1.13 (0.46-2.81, p=0.790)	1.20 (0.48-2.98, p=0.692)
Lower third of esophagus	0.91 (0.37-2.24, p=0.846)	0.93 (0.38-2.29, p=0.882)
T stage		
T1	Ref	Ref
T2	1.30 (0.93-1.82, p=0.124)	1.41 (1.02-1.96, p=0.037)
T3	1.75 (1.29-2.39, p<.001)	1.99 (1.49-2.65, p<.001)
Chemotherapy		
Yes	Ref	Ref
No	0.64 (0.48-0.86, p=0.003)	0.72 (0.53-0.98, p=0.034)
LONT		
<1.0	Ref	
1.0-2.3	0.70 (0.57-0.86, p=0.001)	
>2.3	0.52 (0.39-0.69, p<.001)	
N stage		
N0		Ref
N1		1.36 (1.12-1.66, p=0.002)
N2		1.66 (1.29-2.14, p<.001)

LONT, Log Odds of Negative Lymph Nodes/T stage ratio.

### Random survival forest analysis

**Figure 3:**
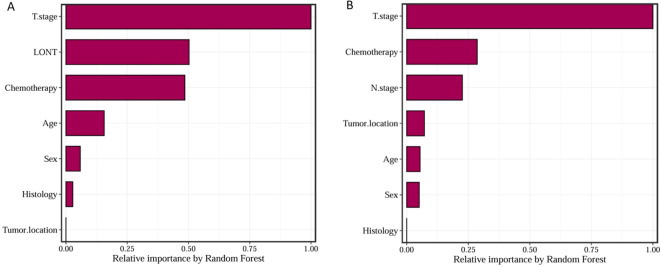
Random Survival Forest model variable relative importance for Model1 **(A)** and Model2 **(B)**.

**Figure 4:**
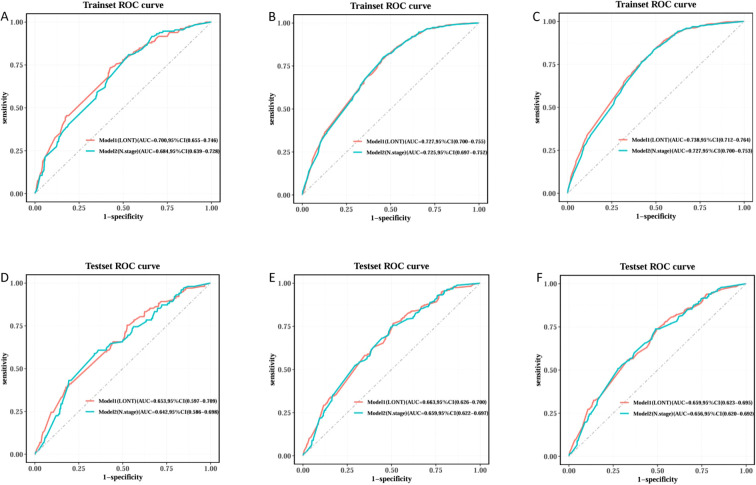
ROC curve comparison of Model1 and Model2 Random Survival Forest model in training and testing sets. Trainset ROC curve in 1 year **(A)**, 3 years **(B)** and 5 years **(C)**. Testing set ROC curve in 1 year **(D)**, 3 years **(E)** and 5 years **(F)**.

**Figure 5:**
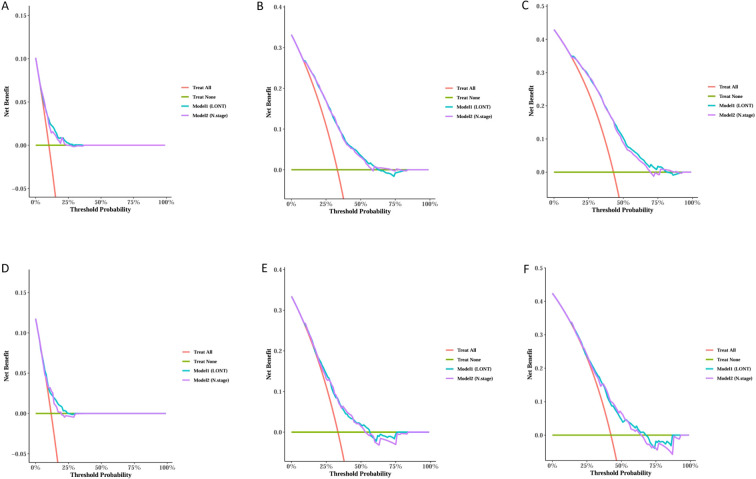
Decision Curve Analysis (DCA) for 1, 3, and 5-years predictions in the Training and Testing sets. Trainset DCA curve in 1 year **(A)**, 3 years **(B)** and 5 years **(C)**. Testing set DCA curve in 1 year **(D)**, 3 years **(E)** and 5 years **(F)**.

**Figure 6:**
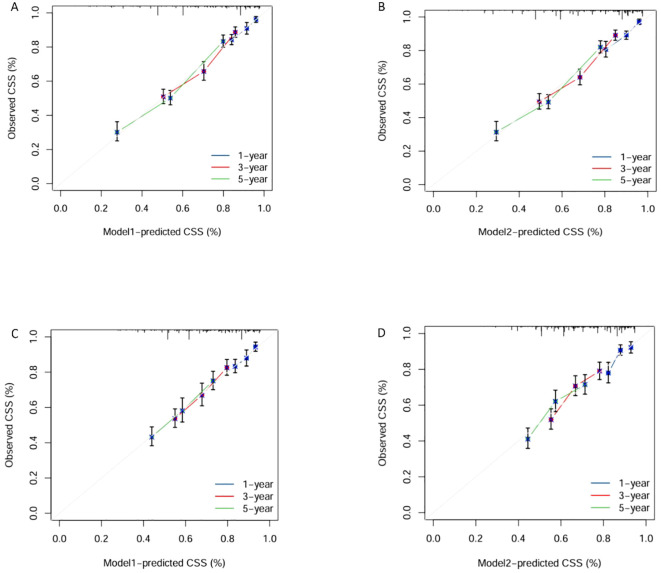
Calibration Plots of Model1 and Model2 for 1, 3, and 5-years survival predictions in the training and testing sets. Calibration plots of training set in Model1 **(A)** and Model2 **(B)**. Calibration plots of testing set in Model1 **(C)** and Model2 **(D)**.

We trained Random Survival Forest (RSF) models using the variables from the two multivariate Cox models: Model 1 (LONT) and Model 2 (N stage). As shown in **Figure 3**, variable importance was visualized for both models. In Model 1, the top three most important variables and their relative importance were T stage (1.00), LONT (0.50), and chemotherapy (0.49). In Model 2, the top three variables were T stage (1.00), chemotherapy (0.29), and N stage (0.23). We further compared the ROC curves of Model 1(LONT) and Model 2(N stage), and found that LONT exhibited superior predictive performance compared to N stage. In the training set, the AUC for Model 1 at 1-, 3-, and 5-years CSS were 0.700, 0.727, and 0.738, respectively, whereas the AUC for Model 2 were 0.684, 0.725, and 0.727, respectively. In the testing set, Model 1 yielded AUC of 0.653, 0.663, and 0.659, whereas Model 2 produced AUC of 0.642, 0.659, and 0.656 for 1-, 3-, and 5-years CSS, respectively (**Figure 4**). To further evaluate the performance of the RSF models, we generated Decision Curve Analysis (DCA) plots (**Figure 5**) and calibration curves (**Figure 6**). The calibration plots showed high consistency between the predicted and observed survival probabilities, with all curves closely aligned with the diagonal line, indicating good calibration of the models. The DCA results demonstrated a higher net benefit across a wide range of threshold probabilities, supporting the clinical utility of the RSF models in individualized risk prediction.

### Survival analysis

**Figure 7:**
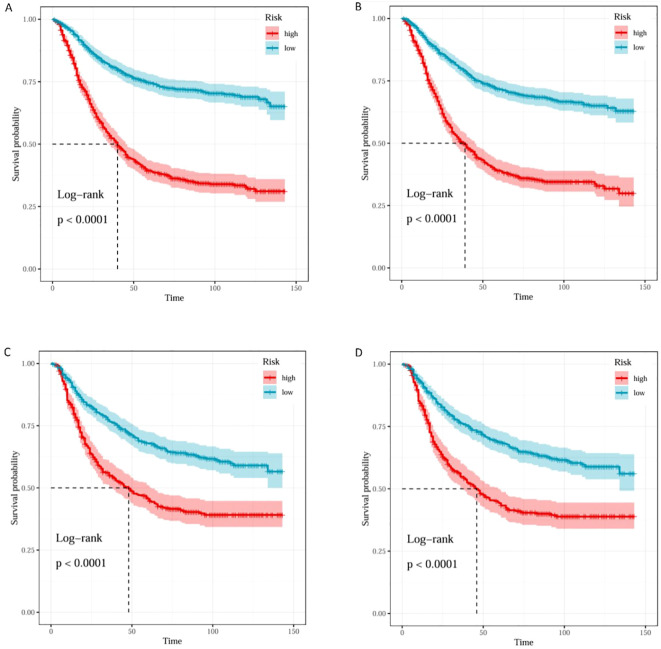
K–M curves of Model1 and Model2 for risk stratification in the training and testing sets. K–M curves of training set in Model1 **(A)** and Model2 **(B)**. K–M curves of testing set in Model1 **(C)** and Model2 **(D)**.

Based on the median predicted risk scores from the RSF models of Model 1(LONT) and Model2(N stage), patients were stratified into high-risk and low-risk groups. Kaplan–Meier survival curves demonstrated that patients in the high-risk group had significantly shorter CSS compared to those in the low-risk group (p < 0.0001) (**Figure 7**).

### Web-based calculator

**Figure 8:**
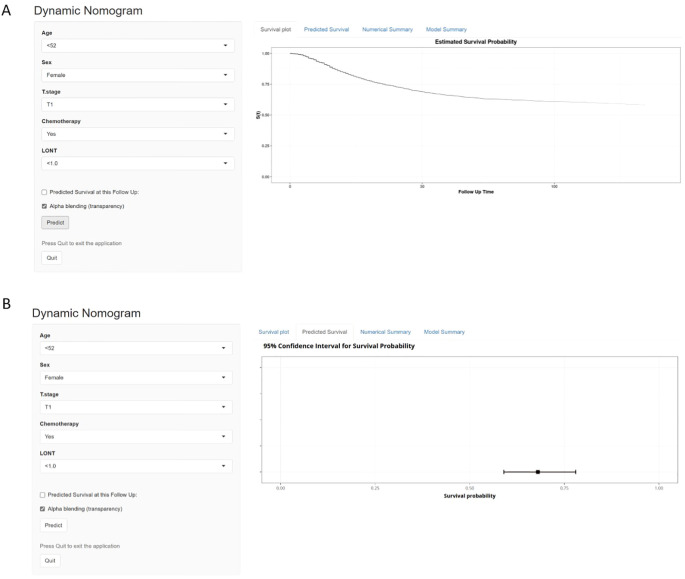
A Web-Based dynamic nomogram calculator. **(A)** displays the predicted survival curve over time. **(B)** provides the estimated survival probability.

We developed an interactive web-based tool incorporating the LONT index, enabling users to input patient-specific clinicopathological variables to rapidly estimate individualized CSS and survival trends (**Figure 8**). This tool was designed to translate our research findings into an intuitive and practical clinical application, providing clinicians with a convenient method for postoperative prognostic assessment and aiding in personalized clinical decision-making. The link to web-based tool is https://hlhmedianaranja.shinyapps.io/dynnomapp/. Patients can access the web-based tool and select either the “Survival Plot” or “Predicted Survival” tab at the top of the page to generate a predicted survival curve or a point estimate of survival probability with a 95% confidence interval, respectively. After entering individual clinical and pathological characteristics using the dropdown menus, users can click the “Predict” button in the lower-left corner to generate the corresponding graphical output(**Supplementary Figure S3**).

## Discussion

In recent years, the log odds of negative lymph nodes/T stage ratio (LONT) has emerged as a novel prognostic marker for evaluating postoperative outcomes in cancer patients, demonstrating significant predictive value (13, 15). Several studies have confirmed a strong association between LONT and tumor progression or prognosis in various cancers (10, 12, 13, 16). However, to our knowledge, no studies have yet explored the prognostic significance of LONT in postoperative esophageal cancer (EC) patients. Therefore, this study investigates the role of LONT as an independent prognostic factor in postoperative EC patients with T1~3N0~2M0 stage, and based on the LONT metric, we developed an interactive web-based tool for dynamic survival prediction.

Increasing attention is being paid to the prognostic significance of negative lymph nodes (NLN) in EC patients. Previous studies have demonstrated that more than three NLNs were associated with improved survival in EC patients (17), while Tang JM identified NRLN > 21 as an independent prognostic factor after surgery in EC patients (18). These findings underscore the potential importance of NLN count in EC prognosis, as it may reflect both the thoroughness of lymphadenectomy and the patient’s immune status. Previous research has also established T stage as a key indicator of tumor aggressiveness and a critical guide for treatment planning (9). Given the prognostic relevance of both NLN and T stage, recent studies have proposed LONT as a novel composite index to better assess the extent of lymph node dissection (LND) and improve individualized prognosis evaluation in cancer patients (12, 13, 16, 19). In the RSF model of this study, although T stage was the strongest predictor, LONT—by incorporating the number of negative lymph nodes—reflects the thoroughness of lymph node dissection and the host immune status, thus providing additional information beyond T stage alone and further improving the model’s predictive performance. Traditional N stage classifies nodal involvement solely based on the number of positive lymph nodes (PLN), without accounting for NLN. As a result, even with identical PLN counts, the total number of examined lymph nodes (ELN) may vary, potentially reflecting differences in actual tumor burden (20). Hence, pN stage may not provide a comprehensive assessment of survival risk. In contrast, LONT combines NLN with T stage, adjusting the NLN ratio according to tumor depth. This allows for a more consistent reflection of lymph node dissection and associated prognostic risk across TNM stage (21). This approach may help address limitations of the TNM stage system and provide a more personalized risk stratification for patients.

In summary, the clinical rationale for LONT is supported on multiple levels. First, the number of NLNs serves as an indirect indicator of the thoroughness and quality of lymphadenectomy. A higher NLN count generally reflects more extensive regional lymph node dissection, which enhances the accuracy of assessing the true metastatic status (22). Second, the NLN count has been recognized as an important prognostic factor for cancer survival. Previous studies suggest that its predictive value may be linked to the host immune response to tumor cells and the molecular characteristics of the cancer itself (23, 24). Moreover, by integrating NLN count with T stage, LONT dynamically adjusts the prognostic weight of NLNs based on tumor invasion depth. This allows for more consistent and reliable risk stratification across different TNM stage (25). Therefore, LONT may overcome the limitations of conventional pN stage, which ignores NLN information. It offers a more comprehensive assessment of prognosis by capturing surgical quality, host immune status, and tumor burden. In this study, LONT outperformed traditional N stage in predicting 1-, 3-, and 5-years cancer-specific survival (CSS) in EC patients, with particularly strong predictive power at the 1-year mark.

This study offers several strengths. First, the study utilizes the Surveillance, Epidemiology, and End Results (SEER) database, covering a large cohort of postoperative EC patients, thereby enhancing the generalizability and statistical power of the findings. Second, it is the first to demonstrate the prognostic value of the LONT metric in EC patients. Third, we developed a dynamic web-based tool for survival prediction to assist clinicians in postoperative follow-up and individualized treatment planning. Naturally, this study also has certain limitations. First, this study is based solely on SEER data, and although internal validation was performed, the lack of external, independent cohort validation limits the generalizability of the model. Future studies should validate the model in external populations and real-world clinical settings to confirm its robustness and clinical applicability. Second, the retrospective nature of the SEER database may introduce selection bias and unmeasured confounders. Third, the SEER database lacks detailed information on chemotherapy, radiotherapy, targeted therapy, and immunotherapy regimens, which may affect patient outcomes. The absence of these treatment-related variables may limit the accuracy of survival prediction and the comprehensive assessment of LONT’s prognostic value. Finally, both our Cox and RSF models demonstrated moderate discriminatory ability, which may limit their utility as standalone clinical tools. Nevertheless, they may still offer practical value when incorporated into a more comprehensive prognostic framework or applied for preliminary risk stratification in large-scale studies.

## Conclusion

Based on the novel prognostic indicator LONT, we developed an interactive web-based tool to predict CSS in postoperative patients with T1~3N0~2M0 esophageal cancer. This online tool provides individualized, quantitative CSS predictions based on patient-specific information, enabling clinicians and patients to better understand disease progression, anticipate treatment outcomes, and set realistic expectations for survival.

## Data Availability

Publicly available datasets were analyzed in this study. This data can be found here: The data used in this study were obtained from the SEER (Surveillance, Epidemiology, and End Results) Program. The data are publicly available and can be accessed via the SEER website. Direct link to the SEER data: (https://seer.cancer.gov/data/). For this study, data were downloaded using the SEERStat software (version 8.4.4), which allows for customized queries and extraction of data from the SEER database.

## Supplementary material

Supplementary
**Supplementary Figure S1** Optimal Age cut-off determination using X-tile software.

Supplementary
**Supplementary Figure S2** Optimal LONT cut-off determination using X-tile software.

Supplementary
**Supplementary Figure S3** User interface of the web tool.
