# Supplementary figures and images for: Prognostic value of the log odds of negative lymph nodes/T stage ratio (LONT) in postoperative esophageal cancer: a SEER-based study
Source: Front Oncol. 2025 Aug 20;15:1619106. doi: 10.3389/fonc.2025.1619106 (PMC12404919; doi:10.3389/fonc.2025.1619106)

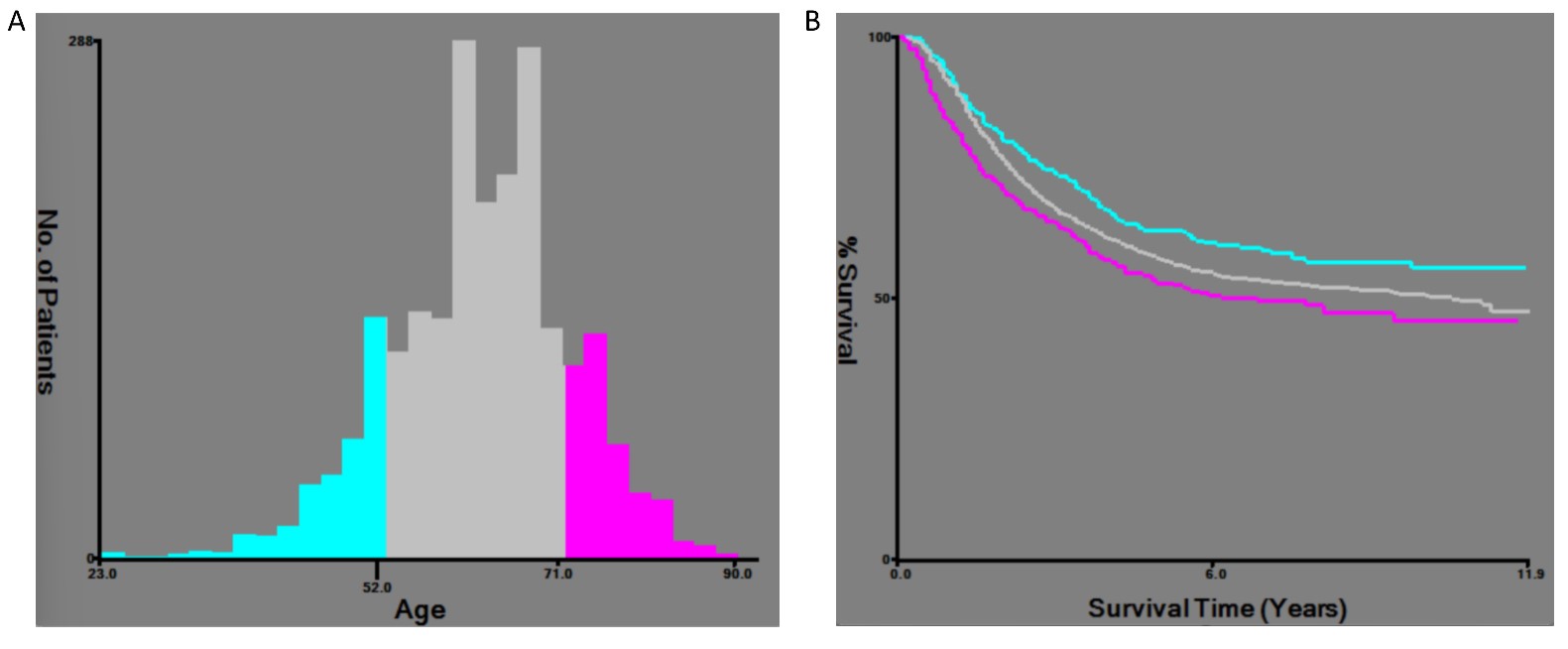

Supplement: Supplementary — Figure S1 Optimal Age cut-off determination using X-tile software. [file Image1.jpeg]

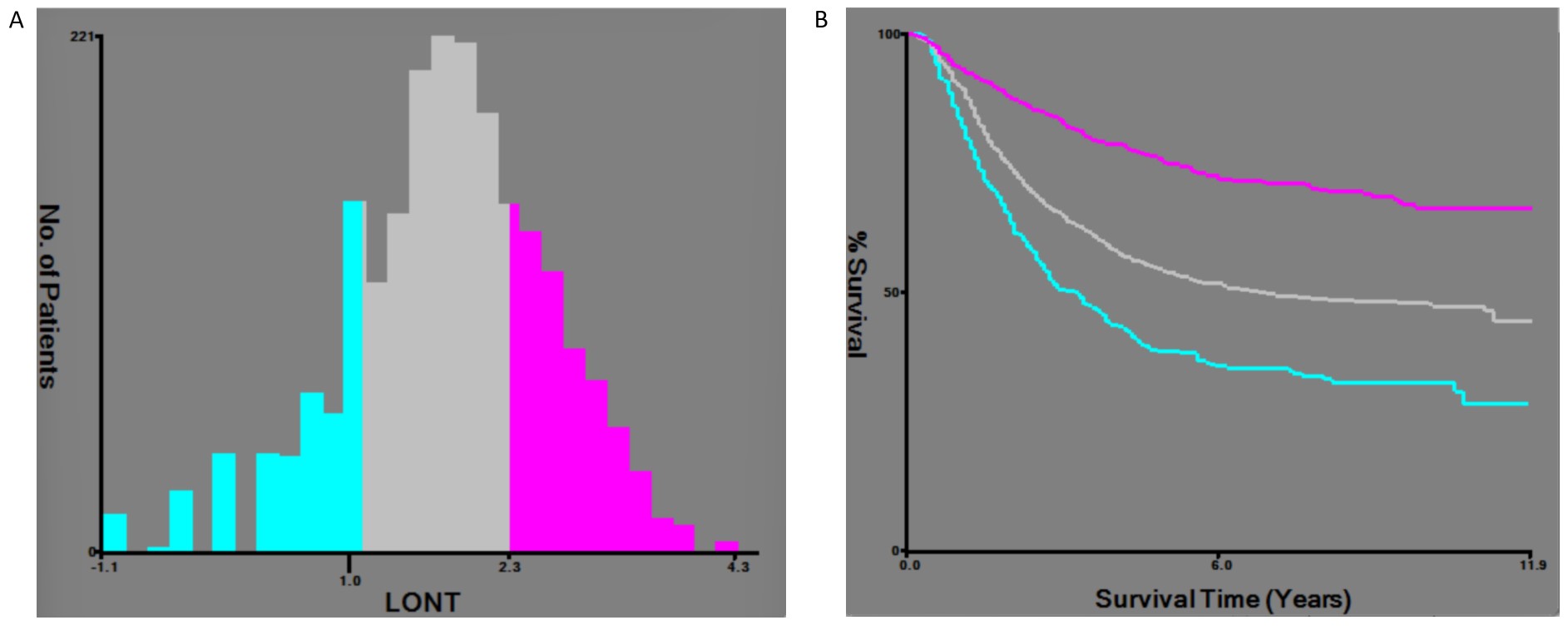

Supplement: Supplementary — Figure S2 Optimal LONT cut-off determination using X-tile software. [file Image2.jpeg]

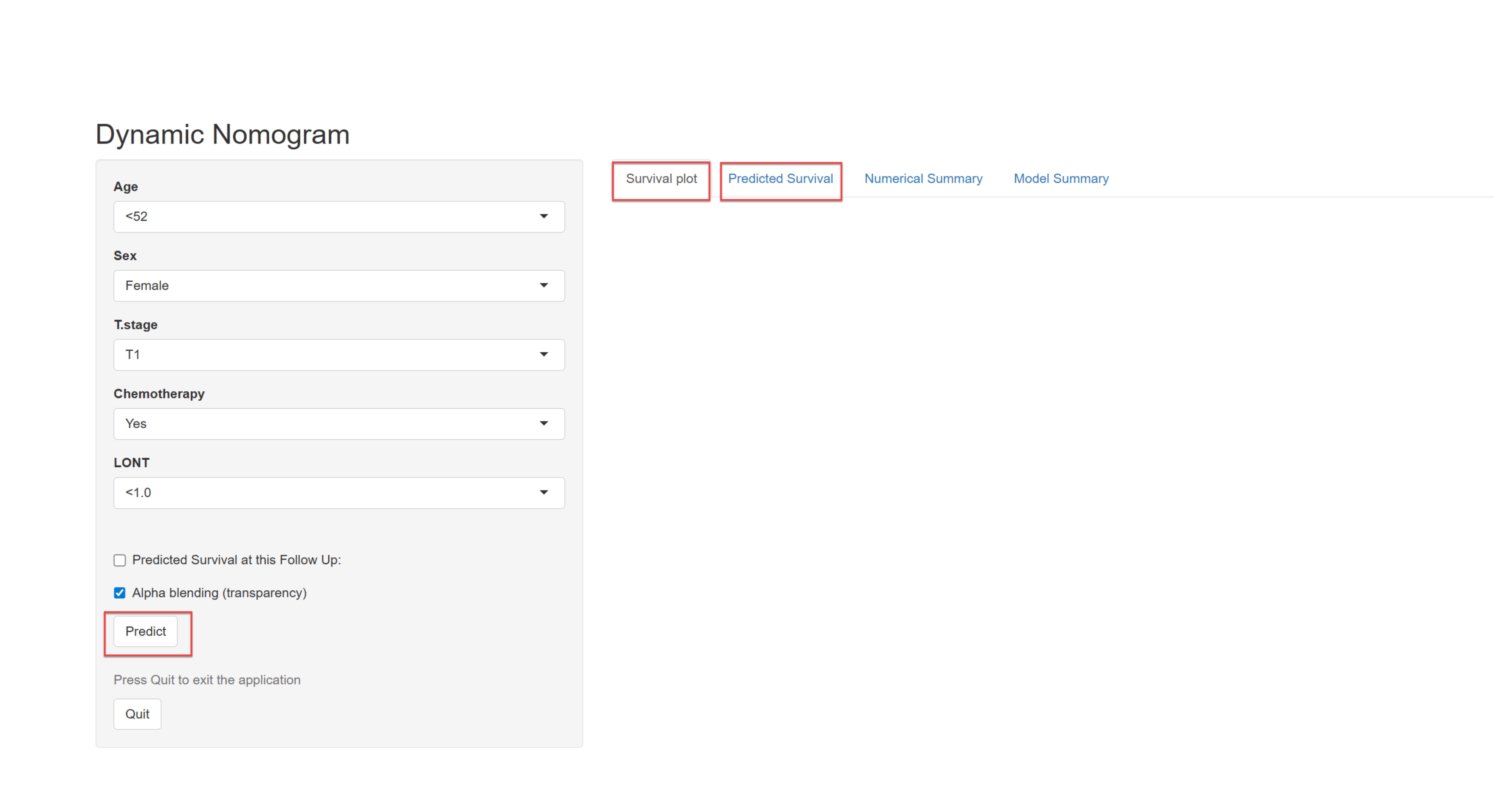

Supplement: Supplementary — Figure S3 User interface of the web tool. [file Image3.jpeg]
